# Association of physical and social neighbourhood environment with movement behaviours among schoolchildren: a compositional data analysis

**DOI:** 10.1186/s12966-026-01879-z

**Published:** 2026-04-08

**Authors:** Ruirui Xing, Jerome N. Rachele, Venurs Loh, Dorothea Dumuid, Željko Pedišić

**Affiliations:** 1 Institute for Health and Sport, Victoria University, Melbourne, Australia; 2 College of Sport, Health and Engineering, Victoria University, Melbourne, Australia; 3 School of Allied Health and Human Performance, College of Health, Adelaide University, Adelaide, Australia; 4 School of Public Health, Li Ka Shing Faculty of Medicine, The University of Hong Kong, Hong Kong, China

**Keywords:** Active travel, Built environment, Crime safety, Land-use mix, Sitting, Social connectedness, Youth

## Abstract

**Background:**

How schoolchildren distribute their time between movement behaviours may be impacted by the neighbourhood environment. Few studies have investigated the associations between the physical and social environment and the full movement behaviour composition, including times spent in moderate-to-vigorous physical activity (MVPA), light physical activity (LPA), sedentary behaviour, and sleep, and their findings are inconsistent. Therefore, our aim was to investigate this association in a large, national-representative sample of schoolchildren from major cities and regional/remote areas.

**Methods:**

We used data from the Longitudinal Study of Australian Children and the Child Health CheckPoint study, collected among 1230 child-parent pairs (child age range: 10–12 years). Parents were asked about neighbourhood general safety, access to destinations and services, and social capital and cohesion. Children’s time spent in MVPA, LPA, sedentary behaviour, and sleep was assessed using wrist-worn GENEActiv accelerometers. The associations between the physical and social environment characteristics (independent variables) and movement behaviour composition expressed as isometric log ratio coordinates (dependent variables) were examined using multiple linear regression analyses, adjusted for age, body mass index, pubertal status, sex, and socioeconomic position.

**Results:**

Among schoolchildren from regional/remote areas, access to destinations and services (Pillai’s trace = 0.030; *p* = 0.010), as well as social capital and cohesion (Pillai’s trace = 0.024; *p* = 0.032) were associated with movement behaviour composition. In specific, better access to destinations and services was associated with higher MVPA and lower LPA, while higher social capital and cohesion were associated with higher MVPA and LPA, and lower sedentary behaviour (with negligible changes in the remaining movement behaviours). We did not find a significant association between general safety and the movement behaviour composition among schoolchildren from regional/remote areas (Pillai’s trace = 0.005; *p* = 0.641) or any significant associations among schoolchildren from major cities (*p* > 0.050 for all).

**Conclusions:**

These findings highlight the importance of access to destinations and services, as well as social capital and cohesion, in shaping the movement behaviour composition among schoolchildren from regional/remote areas. More research is needed to draw conclusions about the association between neighbourhood environment and movement behaviour composition among schoolchildren from major cities.

**Supplementary Information:**

The online version contains supplementary material available at 10.1186/s12966-026-01879-z.

## Introduction

The way schoolchildren use time is important for their health [1, 2]. For example, spending more time in physical activity and sleep at the expense of sedentary behaviour is favourably associated with adiposity status, motor skills, and mental health [1]. To inform the development of health promotion strategies targeting schoolchildren, it is important to understand correlates and determinants of different time-use compositions in this age group. A time-use composition that has received particularly high attention in recent studies consists of movement behaviours, including moderate-to-vigorous physical activity (MVPA), light physical activity (LPA), sedentary behaviour, and sleep [3].

How schoolchildren distribute their time between movement behaviours may be impacted by the neighbourhood environment [4–6]. For example, a recent systematic review found that school proximity and access to sports and recreational facilities, parks, and/or playgrounds are positively associated with different types of physical activity among both children and adolescents, whereas evidence of such associations for access to public transport, good street lighting, and presence of crossing guards was found only among children [6]. Another systematic review found that lower neighbourhood safety and higher neighbourhood noise are associated with poorer sleep outcomes in this age group [5]. However, no such evidence was found for sedentary behaviour [4]. Importantly, almost all studies on these associations have examined each of the movement behaviours individually, without taking into account they are co-dependent parts of the movement behaviour composition [6].

Only a few studies have investigated the associations between the neighbourhood environment and the full movement behaviour composition, including times spent in physical activity, sedentary behaviour, and sleep. A study among Canadian girls aged 8–10 years at a high risk of obesity found that neighbourhood walkability was associated with higher MVPA (relative to LPA, sedentary behaviour, and sleep) [7]. Another Canadian study was conducted among children aged 10–13 years and analysed 29 neighbourhood environment characteristics, encompassing different aspects of walkability, traffic safety, dedicated and non-dedicated play spaces, noise, and lighting, in relation to the movement behaviour composition [8]. They found the following associations: a higher number of cul-de-sacs per km^2^ with higher sedentary behaviour; larger yard spaces with lower MVPA; and higher *non-dedicated play spaces index* with higher LPA, all relative to the remaining movement behaviours [8]. A Singaporean study conducted among children aged 5–8 years did not find significant associations of the availability of facilities for physical activity, environmental facilitators for active mobility, and environmental barriers for active mobility with the movement behaviour composition [9]. Given the limited number of studies and their mixed findings, there is a need to further investigate the association between the neighbourhood environment and movement behaviour composition.

Furthermore, systematic reviews found that previous studies have primarily focused on exploring how the associations between neighbourhood environment and time use vary by sex [10] and socioeconomic status (SES) [11]. A systematic review by Gemmell and colleagues found no consistent moderation effect of gender on the association between built environment features and outdoor free play [10]. A systematic review by Andersen and colleagues found that evidence on socioeconomic moderation has also been mixed, with five studies reporting stronger associations among those with higher SES, seven studies reporting stronger associations among those with lower SES, and fourteen studies reporting no statistically significant moderation effect [11]. Considerably less attention has been given to potential variations between rural and urban settings. Although some previous studies found that associations between neighbourhood environment characteristics and movement behaviours can differ in rural and urban areas [12, 13]. none of them has considered the full movement behaviour composition. More empirical evidence on distinct neighbourhood environment correlates of time use in urban and rural areas is needed, to facilitate and inform the initiatives tailored to specific settlement types.

Therefore, the aim of this study was to investigate the association between neighbourhood environment characteristics and movement behaviour composition consisting of MVPA, LPA, sedentary behaviour, and sleep, in a large, national-representative sample of schoolchildren from major cities and regional/remote areas.

## Methods

### Study design and participants

This was a prospective cohort study, without repeated measurements of exposure and outcome variables. We used data on exposure variables (neighbourhood environment characteristics) from wave 6 of the Longitudinal Study of Australian Children (LSAC) collected in 2014 and data on the outcome variables (movement behaviours) from the Child Health CheckPoint study collected from 2015 to 2016 among a subset of LSAC participants [14]. In wave 1, LSAC included a nationally representative birth cohort of 5107 infants alongside their parents/guardians recruited using a two-stage random sampling design [15], of whom 3764 child-parent pairs (child age range: 10–11 years) remained in wave 6 of the study. The Child Health CheckPoint study included 1874 child-parent pairs (child age range: 11–12 years) who agreed to participate in additional measurements between waves 6 and 7 of LSAC. Our analyses were restricted to the Child Health CheckPoint study participants, including a subsample of 1230 child-parent pairs for whom children’s movement behaviour data were collected using accelerometers.

### Characteristics of neighbourhood environment

In wave 6 of LSAC, parents were asked to express their level of agreement with the following statements about the neighbourhood environment: (1) “This is a safe neighbourhood.”; (2) “It is safe for children to play outside during the day.”; (3) “There is heavy traffic on my street or road.”; (4) “There are good parks, playgrounds and play spaces in this neighbourhood.”; (5) “There is access to close, affordable, regular public transport in this neighbourhood.”; (6) “There is access to basic shopping facilities in this neighbourhood.”; (7) “There is access to basic services such as banks, medical clinics, etc. in this neighbourhood.”; (8) “Most people in your neighbourhood can be trusted.”; (9) “This is a close-knit neighbourhood.”; (10) “People in this neighbourhood generally don’t get along with each other.”; and (11) “People in this neighbourhood do not share the same values.” Participants responded on a Likert scale with four and five points for items 1–8 and items 9–12, respectively.

Three summary scores were calculated from the individual items, including “general safety” (items 1–3; Cronbach alpha [*α*] = 0.494), “access to destinations and services” (items 4–8; *α* = 0.821), and “social capital and cohesion” (items 9–12; *α* = 0.750). This is aligned with the categorisation of neighbourhood environment characteristics by Xing and colleagues [6], proposed based on the factor structure of the Neighbourhood Environment Walkability Scale (NEWS) items [16] and a synthesis of additional neighbourhood characteristics analysed in 149 studies on parental perceptions of neighbourhood environment and physical activity among children and adolescents. We calculated the summary scores, as they are more likely to approximate the normal distribution than the respective individual (ordinal-scale) items. Due to different response scales, the theoretical range of summary scores for access to destinations and services and general safety (i.e. four points) did not match the theoretical range of summary scores for social capital and cohesion (i.e. five points). Therefore, we linearly transformed the summary scores for access to destinations and services and general safety to a scale from 1 to 5, to make them directly comparable with the summary scores for social capital and cohesion.

### Accelerometry and activity logs

Time spent in physical activity, sedentary behaviour, and sleep was assessed using wrist-worn GENEActiv accelerometers (Activinsights, Kimbolton, UK). The participants were asked to wear the accelerometer for eight days, 24 h per day. A day of accelerometry was considered as valid, if it included less than 6 h of non-wear time [17], with non-wear defined according to the criteria proposed by van Hees and colleagues [17]. Visual inspection of accelerometer data and activity logs were used to help determine sleep and non-wear periods. As detailed elsewhere, if the device was removed during sports activities, the non-wear period was filled with 50% MVPA, 30% LPA and 20% sedentary time [18]. Validated cut points (linearly adjusted to account for the 50 Hz sampling frequency) were applied to classify the 60-s epochs into energy expenditure bands (sedentary time, LPA and MVPA) [19]. A day was considered valid if it had > 10 h waking wear time, > 200 min of sleep and < 1000 min of sedentary time. To be included in these analyses, participants were required to have at least four valid days (including one weekend day). Time use across the available days was averaged and weighted for weekdays vs. weekend days at the 5:2 ratio. Data on MVPA, LPA, sedentary behaviour, and sleep were expressed in minutes per day, forming a 4-part time-use composition.

### Sociodemographic characteristics and body mass index

Sociodemographic characteristics and body mass index (BMI) of child participants were assessed at LSAC wave 6. Data on sex and age (years) were obtained from linkage with Medicare, the Australian publicly funded health care insurance scheme. Pubertal status was determined from parental responses to questions about physical signs associated with pubertal maturation of their child using the Pubertal Development Scale [20], and categorised as pre-puberty, early puberty, mid-puberty, and late puberty [21]. Socioeconomic position (z-score) of the household was derived from parental education, income, and occupation [22]. The participants’ main place of residence was classified according to its postcode as “major city” or “regional/remote” (including inner regional, outer regional, remote, and very remote areas) [23]. BMI (weight[kg]/(height[m]^2^) was derived from weight measured using TANITA Body Composition Monitor (TANITA Corporation, Tokyo, Japan) and height measured using BOSCH Professional Laser Measure (Robert Bosch GmbH, Gerlingen, Germany). The measures were taken during a home visit, with participants wearing light clothing and no shoes. BMI was expressed as an age- and sex- specific z-score determined based on the Centers for Disease Control and Prevention growth charts [24].

### Statistical analysis

Absolute frequencies and percentages were calculated for child sex, pubertal status categories, and responding parent sex. Arithmetic means and standard deviations were calculated for child age, child BMI, responding parent age, household socioeconomic position, and characteristics of neighbourhood environment. Compositional means were calculated for the movement behaviour composition. Differences in categorical and numeric variables by the place of residence were tested using the chi-square test and *t*-test for independent samples, respectively. The results were presented for the whole sample and separately for major cities and regional/remote areas.

Compositional data analysis (CoDA) was used to determine the associations between neighbourhood environment characteristics and movement behaviour composition. This process included four steps. First, the 4-part movement behaviour compositions from accelerometry (including MVPA, LPA, sedentary behaviour, and sleep) was expressed by three isometric log ratios (*ilr*) using the *compositions* package [25]. Prior to calculating *ilr* coordinates, the movement behaviour composition was checked for zero values, but no zeros in the dataset were identified. Second, a set of multiple linear regression analyses were conducted, with a neighbourhood environment summary score as an independent variable and the *ilr* coordinates from movement behaviour composition as dependent variables [26]. The models were stratified by place of residence and adjusted for age, body mass index, pubertal status, sex, and socioeconomic position. The adjustments for confounding were based on previous studies on the association between neighbourhood environment characteristics and movement behaviours [6, 27–29]. Third, the models were used to estimate the *ilr* coordinates across sequential increments (0.5) of the neighbourhood environment summary scores. Fourth, the inverse *ilr* transformation was applied to calculate the respective movement behaviour compositions from the estimated *ilr* coordinates. The movement behaviour composition estimates were bootstrapped with 1000 replicates to obtain 95% confidence intervals (CI). The estimates and their 95% bootstrap confidence intervals were plotted to aid interpretation. The results were presented separately for participants from major city and regional/remote areas. All analyses were carried out using R Software version 4.3.2 [30].

## Results

### Sample characteristics

The schoolchildren were on average 11 years old (standard deviation [SD] = 0.3; Table 1). There was almost equal representation of girls (49.3%) and boys (50.7%) and somewhat lower representation of pre-pubertal children (41.5%), compared with children in early, mid, and late puberty adolescents (58.5%). The average BMI was slightly higher than the 50th percentile on the growth chart (z-score = 0.2; SD = 1.0). The parent participants were on average 42.6 years old (SD = 4.8), and almost all of them (97.0%) were females. At the time of the study, most participants lived in major cities (69.2%), and their mean household socioeconomic position score was slightly above the national average (z-score = 0.2; SD = 1.0). Children from major cities had somewhat higher household socioeconomic position *z*-score, compared with children from regional/remote areas (*p* < 0.001). While the differences between the major city sample and regional/remote sample in child age and responding parent age were statistically significant (*p* < 0.010 for both), they were practically negligible. We did not find significant differences between the major city sample and regional/remote sample in the remaining sociodemographic variables and BMI.

**Table 1:** Sample characteristics

Characteristic	Major city sample	Regional/remote sample
	(*n* = 852)	(*n* = 378)
Child sex: female, *n* (%)	414 (48.6)	193 (51.1)
Child age, mean (SD) in years	11.0 (0.3)	10.9 (0.3)
Child pubertal status, *n* (%)		
Pre-puberty	346 (40.6)	165 (43.7)
Early puberty	195 (22.9)	74 (19.6)
Mid-puberty	289 (33.9)	128 (33.9)
Late puberty	22 (2.6)	11 (2.9)
Child body mass index z-score, mean (SD)	0.2 (1.0)	0.3 (0.9)
Responding parent sex: female, *n* (%)	825 (97.0)	365 (96.6)
Responding parent age, mean (SD) in years	43.0 (4.7)	42.0 (5.0)
Household socioeconomic position z-score, mean (SD)	0.4 (1.0)	-0.1 (0.9)
Neighbourhood environment summary score, mean (SD)		
General safety	3.9 (0.7)	4.1 (0.7)
Access to destinations and services	4.2 (0.7)	3.4 (0.9)
Social capital and cohesion	3.7 (0.6)	3.7 (0.7)
Movement behaviour composition (min/day), compositional mean		
Moderate-to-vigorous physical activity	52.5	55.4
Light physical activity	244.6	263.2
Sedentary behaviour	564.8	546.7
Sleep	578.1	574.8

Compositional mean was calculated by linearly adjusting the geometric means for movement behaviours to collectively sum to 1440 min/day; A higher household socioeconomic position is indicated by a higher z-score; SD, standard deviation

On average, parents from major cities rated the access to destinations and services higher and general safety lower than parents from regional/remote areas. The average scores for social capital and cohesion were almost the same among participants from major cities and regional/remote areas.

Schoolchildren from major cities and regional/remote areas spent most of their time in sleep and sedentary behaviour and less time in physical activity. Compared with schoolchildren from regional/remote areas, schoolchildren from major cities had on average somewhat higher sedentary time (565 vs. 547 min/day) and somewhat lower LPA (245 vs. 263 min/day). The mean times spent in the remaining two parts of the movement behaviour composition were similar among schoolchildren from major cities (53 min/day for MVPA and 578 min/day for sleep) and regional/remote areas (55 min/day for MVPA and 575 min/day for sleep).

### Associations between neighbourhood environment and movement behaviour composition

**Fig. 1:**
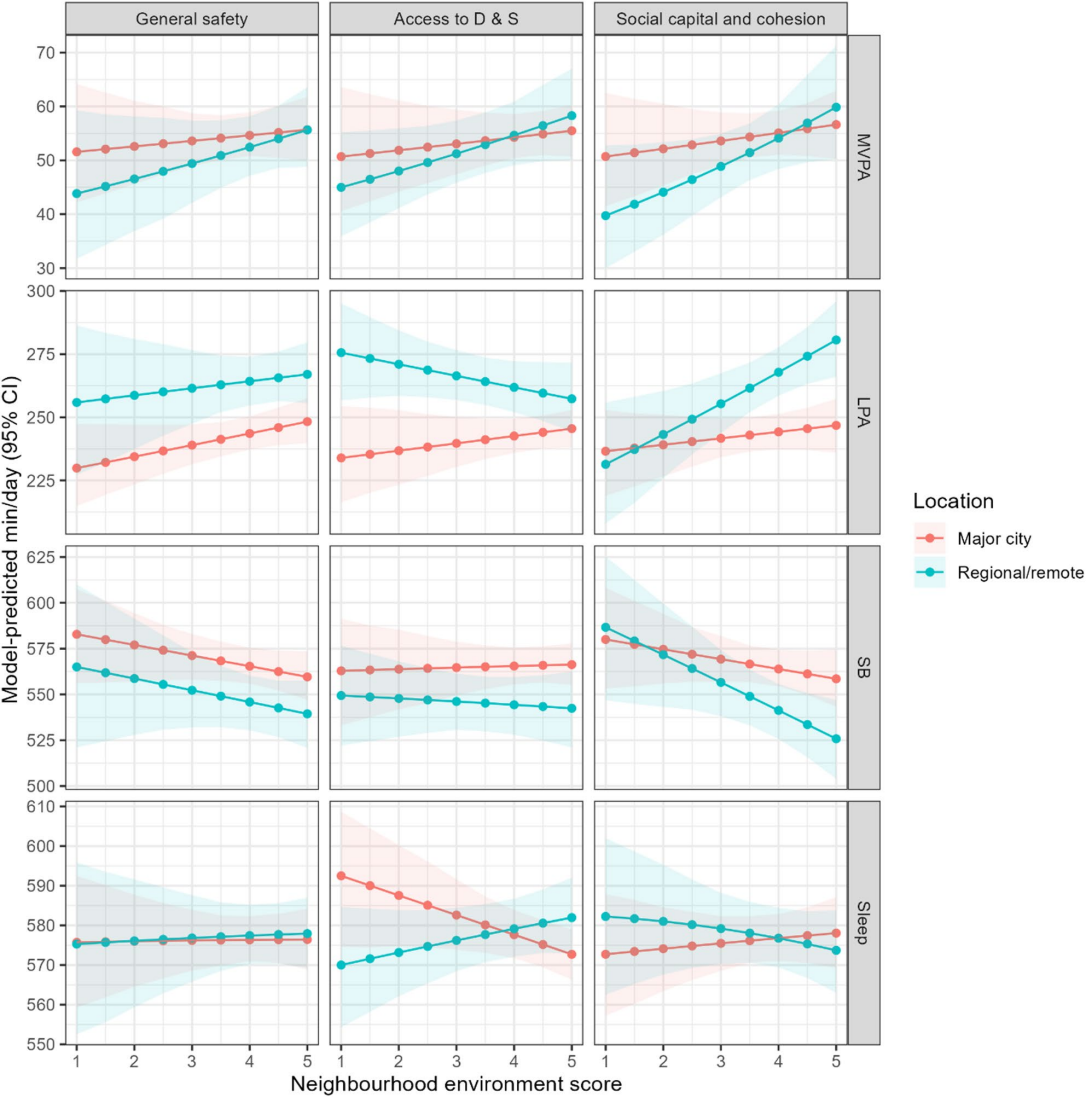
Associations between neighbourhood environment and movement behaviour composition. Notes: Access to D & S, access to destinations and services; CI, confidence interval; LPA, light physical activity; MVPA, moderate-to-vigorous physical activity; SB, sedentary behaviour; Analyses were adjusted for age, body mass index, pubertal status, sex, and socioeconomic position

Among schoolchildren from regional/remote areas, the movement behaviour composition was associated with the access to destinations and services (Pillai’s trace = 0.030; *p* = 0.010; Table 2) and social capital and cohesion (Pillai’s trace = 0.024; *p* = 0.032). In specific, better access to destinations and services was associated with more MVPA, less LPA, and negligible differences in sedentary behaviour and sleep duration, while higher social capital and cohesion were associated with more MVPA and LPA, less sedentary behaviour, and negligible difference in sleep duration (Fig. 1 and Additional file 1). The lowest summary score for access to destinations and services was associated with 45 min/day (95% CI: 36, 55) of MVPA, 276 min/day (95% CI: 257, 295) of LPA, 549 min/day (95% CI: 522, 577) of sedentary behaviour, and 570 min/day (95% CI: 554, 585) of sleep, while the highest score was associated was associated with 58 min/day (95% CI: 50, 67) of MVPA, 257 min/day (95% CI: 244, 272) of LPA, 542 min/day (95% CI: 521, 562) of sedentary behaviour, and 582 min/day (95% CI: 573, 592) of sleep. The lowest summary score for social capital and cohesion was associated with 40 min/day (95% CI: 30, 53) of MVPA, 231 min/day (95% CI: 208, 256) of LPA, 587 min/day (95% CI: 547, 625) of sedentary behaviour, and 582 min/day (95% CI: 563, 602) of sleep, while the highest score was associated was associated with 60 min/day (95% CI: 50, 71) of MVPA, 281 min/day (95% CI: 266, 296) of LPA, 526 min/day (95% CI: 504, 547) of sedentary behaviour, and 574 min/day (95% CI: 563, 584) of sleep. In this subsample, we did not find a significant association between general safety and the movement behaviour composition (Pillai’s trace = 0.005; *p* = 0.641).

These associations have been confirmed in our sensitivity analyses that included only schoolchildren who did not change the type of their place of residence (e.g., by reallocating from a regional/remote area to a major city) between LSAC wave 6 and Child Health CheckPoint study data collections (Additional file 2).

Among schoolchildren from major cities, we did not find any significant associations between the neighbourhood environment and movement behaviour composition (*p* > 0.050 for all associations).

**Table 2:** Associations between neighbourhood environment and movement behaviour composition: results from compositional MANOVAs

Independent variable	Major city sample (*n* = 852)			Regional/remote sample (*n* = 378)		
	Pillai’s trace	F	*p*	Pillai’s trace	F	*p*
Model 1						
General safety	0.003	0.9	0.437	0.005	0.6	0.641
Model 2						
Access to destinations and services	0.005	1.5	0.211	0.030	3.8	0.010
Model 3						
Social capital and cohesion	0.002	0.5	0.702	0.024	3.0	0.032

The multivariate models also included age, body mass index, pubertal status, sex, and socioeconomic position as independent variables

## Discussion

### Key findings

The key findings for children from regional/remote areas are that: (1) better access to destinations and services is associated with more MVPA, less LPA, and negligible differences in the remaining movement behaviours; and (2) higher social capital and cohesion are associated with more MVPA and LPA, less sedentary behaviour, and negligible difference in sleep duration. We did not find a significant association between general safety and movement behaviour composition among children from regional/remote areas. We also did not find any significant associations between any of the three neighbourhood environment characteristics and movement behaviour composition among children from major cities.

### General safety

We did not find a significant association between general safety and movement behaviour composition, which is in line with the findings of a previous CoDA-based study [8] and a recent systematic review [6]. Interestingly, the review found that two traffic safety variables (namely, good street lighting and presence of crossing guards) are associated with more active travel among children [6]. Therefore, it might be that only specific safety-related characteristics of neighbourhood environment are associated with some types of physical activity, such as active travel. However, no conclusions about such specific associations could be drawn in the current study, because our analyses included a summary measure of general safety (i.e. a combination of crime/personal and traffic safety) and non-type-specific physical activity variables. The results of our study are not consistent with previous findings on neighbourhood environment characteristics associated with sedentary behaviour and sleep. For example, a systematic review by Parajára and colleagues [31] found that general and crime safety are negatively associated with sedentary behaviour, while a systematic review by Mayne and colleagues [5] found that these neighbourhood environment characteristics are generally associated with poorer sleep outcomes. None of the individual studies included in these reviews has analysed the whole movement behaviour composition using CoDA, which may explain the differences in findings.

### Access to destinations and services

Unlike most previous studies that analysed MVPA as a standalone variable [6] and two studies that considered MVPA as part of the movement behaviour composition [8, 9], we found a favourable association between access to destinations and services and MVPA (as a part of the movement behaviour composition), albeit only among children in regional/remote areas. The discrepancy between findings could be due to differences in analytical samples. While our finding refers exclusively to schoolchildren in regional/remote areas, most previous studies included either urban or mixed samples [8, 9]. In our sample, the access to destinations and services was worse and less homogenous among children from regional/remote areas, compared with children from urban areas. It may be that children from regional/remote areas who live closer to destinations and service such as school, playgrounds, and sports clubs are encouraged to engage in more active travel (which then increases their MVPA level), compared with those who live further away from such destinations and services. This would be in accordance with the most consistent find of previous studies, indicating that schoolchildren who live closer to schools are more likely to engage in active travel [6]. It could be that the variability in access to destinations and services among children from urban areas is not large enough to be reflected in their movement behaviour composition.

In contrast to Campbell and Janssen who found a positive association between non-dedicated spaces index and LPA [8], we found a negative association between access to destinations and services and LPA (as a part of the movement behaviour composition). The reason for the contrasting findings may be that the non-dedicated spaces index used in the above-mentioned study included park area, non-park greenspace area, yard size, and cul-de-sacs, while the measure of access to destinations and services used in our study referred to parks, playgrounds, play spaces, public transport shopping facilities, and basic services such as banks, medical clinics. It could be that some aspects of access to destinations and services are favourably associated while others are unfavourably associated with LPA. The ratio of children’s MVPA and LPA seems to be higher (i.e. they spend more time in MVPA relative to LPA) in settings outside their home (e.g. on playgrounds and play spaces), than at home [32]. Given that the availability of recreational facilities, play spaces, and playgrounds in the neighbourhood is favourably associated with the time spent outdoors [33], this could explain why children with better access to destinations and services spend more time in MVPA and less time in LPA.

### Social capital and cohesion

Our findings on the association of social capital and cohesion with MVPA and sedentary behaviour (as parts of the movement behaviour composition) in regional/remote areas are consistent with previous research [34, 35]. For example, a recent American study found that higher social capital and cohesion (e.g. knowing where to go for help and neighbours watching out for each other) are associated with a higher likelihood of children meeting physical activity recommendations, that is, at least 60 min of MVPA per day [35]. In another American study, higher informal social control was associated with higher MVPA and lower screen time [34]. However, these comparisons should be taken with caution, because previous studies did not examine the whole time-use composition and their measures of social capital and cohesion were different from the ones used in our study.

When parents have trust in their neighbours and experience a sense of community belonging, they are more inclined to encourage and permit their children to play with others and utilize shared spaces that are typically out of home [36]. Given that children are least physically active while being at home [32], this could explain why higher social capital and cohesion are associated with more MVPA and LPA and less sedentary behaviour.

A previous study found a stronger association between social cohesion and walking to school among children from large towns, compared with children from small towns [37]. In contrast, from our findings, it seems that the association is stronger in regional/remote area, compared with major cities. This discrepancy in findings could be due to differences between American and Australian social and behavioural contexts.

### Implications for policy and practice

To improve the health of schoolchildren, public health interventions and policies should focus on neighbourhood characteristics that generally support movement behaviour compositions with higher proportion of MVPA and lower proportion of sedentary behaviour. Our findings suggest that improving access to destinations and services and increasing social capital and cohesion should be priorities in this endeavour, particularly for children living in regional/remote areas. For example, in terms of built environment, the national and local governments should consider improving access to public transport and building more parks, playgrounds, and play spaces. In terms of social environment, it is important to build trust between neighbours, create better inter-personal relationships, and support diversity and inclusion within communities. This could result in reallocations of time from sedentary behaviour to MVPA among schoolchildren, which would be favourable for several important health outcomes, such as adiposity status, physical fitness, and mental health [1].

We found that the highest access to destinations and services is associated with 13 min/day higher MVPA and 19 min/day lower LPA than the lowest access to destinations and services, while the highest social capital and cohesion was associated with 20 min/day higher MVPA, 50 min/day higher LPA, and 61 min/day lower sedentary behaviour than the lowest access to destinations and services. The magnitude of these estimated differences can be considered as practically significant for health promotion purposes, given that 13 min/day is equivalent to 22.0% and 20 min/day is equivalent to 33.3% of the recommended minimum daily duration of MVPA for health among schoolchildren [38, 39].

### Strengths and limitations

This study had several methodological strengths. First, it was conducted in a large sample, covering various sociodemographic groups and different types of neighbourhood environment. Second, movement behaviours were assessed using accelerometers, enabling continuous data collection 24 h per day. Third, the use of CoDA enabled us to adequately address the compositional properties of movement behaviour data and simultaneously consider all parts of the time-use composition in the analysis.

Several limitations should also be considered. First, the study design limited conclusions we could make about causal relationships, as the dataset we used does not include repeated measurements of either exposure or outcome variables. There is a possibility of reverse causation. For example, it is possible that some parents started perceiving their neighbourhood as being safe, because their children spent a lot of time in (outdoor) physical activity, without any safety-related issues. Second, the neighbourhood environment characteristics were reported by parents, whose perceptions of neighbourhood environment may not be entirely accurate. Parent-reported data on neighbourhood characteristics may not necessarily reflect the true features of the environment. However, some neighbourhood environment characteristics, such as social capital and cohesion, would be very challenging (if not impossible) to capture using objective measures. Third, our analyses encompassed only three aspects of neighbourhood environment, while some other, potentially relevant neighbourhood environment characteristics (e.g. physical barriers, greenery and aesthetics, residential density) were not considered. Fourth, given that we analysed only the 4-part movement behaviour composition consisting of MVPA, LPA, sedentary behaviour, and sleep, conclusions could not be made for other important variables, such as overall physical activity, vigorous physical activity, moderate physical activity, bed time, screen time, and domain-specific components of time use. Fifth, due to attrition of participants between LSAC wave 1 and Child Health CheckPoint study, as well as potential temporal changes in the sociodemographic structure of the study population, our analytic sample may not be fully nationally representative, which may have introduced selection bias and reduced the generalisability of our findings.

## Conclusions

Among children from regional/remote areas, we found that better access to destinations and services is associated with more MVPA and less LPA, while higher social capital and cohesion are associated with more MVPA and LPA and less sedentary behaviour. To promote healthier use of time among schoolchildren from regional/remote areas, public health policies and interventions should focus on improving access to destinations and services and increasing social capital and cohesion in neighbourhoods.

More research is needed to draw conclusions about the association between neighbourhood environment and movement behaviour composition, especially among schoolchildren from major cities. Future studies should consider using longitudinal design with repeated measurements of both exposure and outcome variables or (quasi)experimental study design, as well as objective measures of neighbourhood environment, while assessing a wider range of neighbourhood environment characteristics. Future studies should also consider analysing other movement behaviour compositions that are relevant for population health.

## Supplementary Information

Supplementary Material 1.

Supplementary Material 2.

## Data Availability

Application via the DSS Longitudinal Studies Dataverse (http://dx.doi.org/10.26193/BAA3N6) is needed to gain access to the datasets used in this study. The data are provided under the license by the Australian Department of Social Services; hence, restrictions may apply to their availability and use.
