# Supplementary material for: Association of physical and social neighbourhood environment with movement behaviours among schoolchildren: a compositional data analysis
Source: Int J Behav Nutr Phys Act. 2026 Apr 8;23:35. doi: 10.1186/s12966-026-01879-z (PMC13063444; doi:10.1186/s12966-026-01879-z)
Supplement: Supplementary file 1 — Supplementary Material 1. [file 12966_2026_1879_MOESM1_ESM.docx]

Additional file 1. Associations between neighbourhood environment and movement behaviour composition: results of multiple linear regression analyses

| **Model 1: General safety** | | | | | | | | | | | | |
| --- | --- | --- | --- | --- | --- | --- | --- | --- | --- | --- | --- | --- |
| **Major city sample (*n* = 852)** | | | | | | | | | | | | |
|  | ***ilr*_1_** | | | | ***ilr*_2_** | | | | ***ilr*_3_** | | | |
|  | **Beta** | **SE** | **t** | ***p*** | **Beta** | **SE** | **t** | ***p*** | **Beta** | **SE** | **t** | ***p*** |
| Intercept | -0.721 | 0.163 | -4.431 | <0.001 | -0.044 | 0.283 | -0.155 | 0.877 | 0.172 | 0.692 | 0.248 | 0.804 |
| General Safety | -0.007 | 0.007 | -1.030 | 0.303 | 0.020 | 0.013 | 1.577 | 0.115 | 0.014 | 0.031 | 0.455 | 0.650 |
| Age | 0.001 | <0.001 | 4.655 | <0.001 | -0.001 | <0.001 | -2.931 | 0.003 | -0.003 | 0.001 | -2.193 | 0.029 |
| Sex | -0.004 | 0.011 | -0.383 | 0.702 | 0.065 | 0.019 | 3.415 | 0.001 | -0.346 | 0.046 | -7.447 | <0.001 |
| SEP | -0.006 | 0.005 | -1.282 | 0.200 | -0.004 | 0.008 | -0.468 | 0.640 | 0.041 | 0.020 | 2.106 | 0.036 |
| Late puberty | 0.048 | 0.031 | 1.532 | 0.126 | -0.079 | 0.054 | -1.458 | 0.145 | -0.290 | 0.133 | -2.185 | 0.029 |
| Mid-puberty | 0.006 | 0.013 | 0.445 | 0.657 | -0.027 | 0.023 | -1.173 | 0.241 | -0.141 | 0.055 | -2.545 | 0.011 |
| Pre-puberty | 0.011 | 0.013 | 0.868 | 0.386 | -0.025 | 0.022 | -1.130 | 0.259 | -0.033 | 0.054 | -0.621 | 0.535 |
| zBMI | 0.013 | 0.005 | 2.717 | 0.007 | 0.010 | 0.009 | 1.133 | 0.258 | -0.071 | 0.021 | -3.398 | 0.001 |
| Multiple R^2^ | 0.044 |  |  |  | 0.035 |  |  |  | 0.142 |  |  |  |
| **Regional/remote sample (*n* = 378)** | | | | | | | | | | | | |
|  | ***ilr*_1_** | | | | ***ilr*_2_** | | | | ***ilr*_3_** | | | |
|  | **Beta** | **SE** | **t** | ***p*** | **Beta** | **SE** | **t** | ***p*** | **Beta** | **SE** | **t** | ***p*** |
| Intercept | -0.467 | 0.241 | -1.940 | 0.053 | 0.234 | 0.411 | 0.569 | 0.569 | 0.664 | 0.902 | 0.736 | 0.462 |
| General Safety | -0.009 | 0.011 | -0.840 | 0.402 | 0.013 | 0.018 | 0.706 | 0.480 | 0.052 | 0.040 | 1.284 | 0.200 |
| Age | 0.001 | <0.001 | 2.165 | 0.031 | -0.002 | 0.001 | -2.596 | 0.010 | -0.004 | 0.002 | -2.342 | 0.020 |
| Sex | -0.039 | 0.018 | -2.129 | 0.034 | 0.085 | 0.031 | 2.747 | 0.006 | -0.357 | 0.068 | -5.254 | <0.001 |
| SEP | -0.008 | 0.008 | -0.933 | 0.351 | 0.007 | 0.014 | 0.488 | 0.626 | 0.022 | 0.031 | 0.718 | 0.473 |
| Late puberty | 0.039 | 0.045 | 0.863 | 0.389 | 0.096 | 0.077 | 1.244 | 0.214 | -0.034 | 0.169 | -0.200 | 0.841 |
| Mid-puberty | 0.024 | 0.021 | 1.160 | 0.247 | 0.009 | 0.036 | 0.246 | 0.806 | 0.031 | 0.078 | 0.392 | 0.695 |
| Pre-puberty | 0.008 | 0.020 | 0.376 | 0.707 | 0.016 | 0.034 | 0.466 | 0.641 | -0.063 | 0.075 | -0.843 | 0.400 |
| zBMI | 0.008 | 0.008 | 1.092 | 0.275 | 0.006 | 0.013 | 0.439 | 0.661 | -0.103 | 0.029 | -3.557 | <0.001 |
| Multiple R^2^ | 0.043 |  |  |  | 0.057 |  |  |  | 0.124 |  |  |  |
|  | | | | | | | | | | | | |
| **Model 2: Access to destinations and services** | | | | | | | | | | | | |
| **Major city sample (*n* = 852)** | | | | | | | | | | | | |
|  | ***ilr*_1_** | | | | ***ilr*_2_** | | | | ***ilr*_3_** | | | |
|  | **Beta** | **SE** | **t** | ***p*** | **Beta** | **SE** | **t** | ***p*** | **Beta** | **SE** | **t** | ***p*** |
| Intercept | -0.771 | 0.161 | -4.800 | <0.001 | 0.011 | 0.280 | 0.040 | 0.968 | 0.188 | 0.683 | 0.275 | 0.783 |
| Access to destinations and services | 0.007 | 0.007 | 1.034 | 0.302 | 0.013 | 0.012 | 1.067 | 0.286 | 0.018 | 0.029 | 0.622 | 0.534 |
| Age | 0.001 | <0.001 | 4.579 | <0.001 | -0.001 | <0.001 | -3.031 | 0.003 | -0.003 | 0.001 | -2.243 | 0.025 |
| Sex | -0.004 | 0.011 | -0.325 | 0.745 | 0.065 | 0.019 | 3.407 | 0.001 | -0.345 | 0.046 | -7.437 | <0.001 |
| SEP | -0.007 | 0.005 | -1.500 | 0.134 | -0.003 | 0.008 | -0.385 | 0.700 | 0.041 | 0.020 | 2.106 | 0.036 |
| Late puberty | 0.051 | 0.031 | 1.637 | 0.102 | -0.081 | 0.054 | -1.496 | 0.135 | -0.289 | 0.132 | -2.183 | 0.029 |
| Mid-puberty | 0.005 | 0.013 | 0.413 | 0.680 | -0.027 | 0.023 | -1.194 | 0.233 | -0.142 | 0.055 | -2.559 | 0.011 |
| Pre-puberty | 0.010 | 0.013 | 0.820 | 0.413 | -0.026 | 0.022 | -1.168 | 0.243 | -0.035 | 0.054 | -0.646 | 0.518 |
| zBMI | 0.013 | 0.005 | 2.746 | 0.006 | 0.009 | 0.009 | 1.107 | 0.269 | -0.071 | 0.021 | -3.404 | 0.001 |
| Multiple R^2^ | 0.044 |  |  |  | 0.033 |  |  |  | 0.142 |  |  |  |
| **Regional/remote sample (*n* = 378)** | | | | | | | | | | | | |
|  | ***ilr*_1_** | | | | ***ilr*_2_** | | | | ***ilr*_3_** | | | |
|  | **Beta** | **SE** | **t** | ***p*** | **Beta** | **SE** | **t** | ***p*** | **Beta** | **SE** | **t** | ***p*** |
| Intercept | -0.448 | 0.245 | -1.832 | 0.068 | 0.355 | 0.418 | 0.849 | 0.396 | 0.389 | 0.913 | 0.426 | 0.670 |
| Access to destinations and services | -0.006 | 0.008 | -0.769 | 0.443 | -0.015 | 0.013 | -1.126 | 0.261 | 0.060 | 0.029 | 2.101 | 0.036 |
| Age | 0.001 | <0.001 | 2.023 | 0.044 | -0.002 | 0.001 | -2.638 | 0.009 | -0.003 | 0.002 | -2.050 | 0.041 |
| Sex | -0.039 | 0.018 | -2.178 | 0.030 | 0.087 | 0.031 | 2.812 | 0.005 | -0.353 | 0.068 | -5.225 | <0.001 |
| SEP | -0.008 | 0.008 | -0.935 | 0.350 | 0.012 | 0.014 | 0.830 | 0.407 | 0.017 | 0.031 | 0.549 | 0.583 |
| Late puberty | 0.043 | 0.045 | 0.953 | 0.341 | 0.097 | 0.077 | 1.267 | 0.206 | -0.066 | 0.168 | -0.391 | 0.696 |
| Mid-puberty | 0.022 | 0.021 | 1.063 | 0.288 | 0.005 | 0.036 | 0.127 | 0.899 | 0.049 | 0.078 | 0.630 | 0.529 |
| Pre-puberty | 0.007 | 0.020 | 0.342 | 0.732 | 0.013 | 0.034 | 0.388 | 0.698 | -0.055 | 0.075 | -0.739 | 0.460 |
| zBMI | 0.008 | 0.008 | 1.099 | 0.273 | 0.006 | 0.013 | 0.428 | 0.669 | -0.103 | 0.029 | -3.577 | <0.001 |
| Multiple R^2^ | 0.043 |  |  |  | 0.059 |  |  |  | 0.130 |  |  |  |
|  | | | | | | | | | | | | |
| **Model 3: Social capital and cohesion** | | | | | | | | | | | | |
| **Major city sample (*n* = 852)** | | | | | | | | | | | | |
|  | ***ilr*_1_** | | | | ***ilr*_2_** | | | | ***ilr*_3_** | | | |
|  | **Beta** | **SE** | **t** | ***p*** | **Beta** | **SE** | **t** | ***p*** | **Beta** | **SE** | **t** | ***p*** |
| Intercept | -0.730 | 0.161 | -4.535 | <0.001 | 0.010 | 0.281 | 0.036 | 0.971 | 0.168 | 0.685 | 0.246 | 0.806 |
| Social capital and cohesion | -0.008 | 0.008 | -1.100 | 0.272 | 0.012 | 0.013 | 0.877 | 0.381 | 0.023 | 0.032 | 0.713 | 0.476 |
| Age | 0.001 | <0.001 | 4.719 | <0.001 | -0.001 | <0.001 | -2.992 | 0.003 | -0.003 | 0.001 | -2.232 | 0.026 |
| Sex | -0.004 | 0.011 | -0.404 | 0.686 | 0.065 | 0.019 | 3.408 | 0.001 | -0.345 | 0.046 | -7.424 | <0.001 |
| SEP | -0.006 | 0.005 | -1.277 | 0.202 | -0.003 | 0.008 | -0.384 | 0.701 | 0.041 | 0.020 | 2.079 | 0.038 |
| Late puberty | 0.049 | 0.031 | 1.569 | 0.117 | -0.083 | 0.054 | -1.527 | 0.127 | -0.291 | 0.132 | -2.198 | 0.028 |
| Mid-puberty | 0.006 | 0.013 | 0.487 | 0.626 | -0.027 | 0.023 | -1.202 | 0.230 | -0.142 | 0.055 | -2.571 | 0.010 |
| Pre-puberty | 0.011 | 0.013 | 0.877 | 0.381 | -0.025 | 0.022 | -1.133 | 0.258 | -0.034 | 0.054 | -0.628 | 0.530 |
| zBMI | 0.013 | 0.005 | 2.707 | 0.007 | 0.010 | 0.009 | 1.123 | 0.262 | -0.071 | 0.021 | -3.388 | 0.001 |
| Multiple R^2^ | 0.044 |  |  |  | 0.033 |  |  |  | 0.142 |  |  |  |
| **Regional/remote sample (*n* = 378)** | | | | | | | | | | | | |
|  | ***ilr*_1_** | | | | ***ilr*_2_** | | | | ***ilr*_3_** | | | |
|  | **Beta** | **SE** | **t** | ***p*** | **Beta** | **SE** | **t** | ***p*** | **Beta** | **SE** | **t** | ***p*** |
| Intercept | -0.443 | 0.241 | -1.841 | 0.066 | 0.129 | 0.408 | 0.316 | 0.752 | 0.558 | 0.900 | 0.620 | 0.536 |
| Social capital and cohesion | -0.017 | 0.011 | -1.561 | 0.119 | 0.052 | 0.018 | 2.862 | 0.004 | 0.084 | 0.040 | 2.088 | 0.037 |
| Age | 0.001 | <0.001 | 2.167 | 0.031 | -0.002 | 0.001 | -2.684 | 0.008 | -0.004 | 0.002 | -2.331 | 0.020 |
| Sex | -0.037 | 0.018 | -2.057 | 0.040 | 0.079 | 0.031 | 2.582 | 0.010 | -0.363 | 0.068 | -5.355 | <0.001 |
| SEP | -0.008 | 0.008 | -1.010 | 0.313 | 0.006 | 0.014 | 0.459 | 0.647 | 0.026 | 0.031 | 0.842 | 0.400 |
| Late puberty | 0.036 | 0.045 | 0.807 | 0.420 | 0.107 | 0.076 | 1.408 | 0.160 | -0.023 | 0.168 | -0.134 | 0.894 |
| Mid-puberty | 0.026 | 0.021 | 1.252 | 0.212 | 0.003 | 0.035 | 0.075 | 0.940 | 0.021 | 0.078 | 0.273 | 0.785 |
| Pre-puberty | 0.010 | 0.020 | 0.480 | 0.631 | 0.010 | 0.034 | 0.293 | 0.770 | -0.074 | 0.075 | -0.986 | 0.325 |
| zBMI | 0.009 | 0.008 | 1.111 | 0.267 | 0.006 | 0.013 | 0.423 | 0.672 | -0.103 | 0.029 | -3.593 | <0.001 |
| Multiple R^2^ | 0.047 |  |  |  | 0.077 |  |  |  | 0.130 |  |  |  |

Notes: Beta, unstandardised regression coefficient; *ilr*, isometric log ratio; *p*, p-value for beta; SE, standard error of beta; SEP, socioeconomic position; t, t-value for beta; zBMI, body mass index z-score
