# Supplementary material for: Association of physical and social neighbourhood environment with movement behaviours among schoolchildren: a compositional data analysis
Source: Int J Behav Nutr Phys Act. 2026 Apr 8;23:35. doi: 10.1186/s12966-026-01879-z (PMC13063444; doi:10.1186/s12966-026-01879-z)
Supplement: Supplementary file 2 — Supplementary Material 2. [file 12966_2026_1879_MOESM2_ESM.docx]

Additional file 2. Associations between neighbourhood environment and movement behaviour composition: sensitivity analysis including only participants whose place of residence type did not change over the course of the study


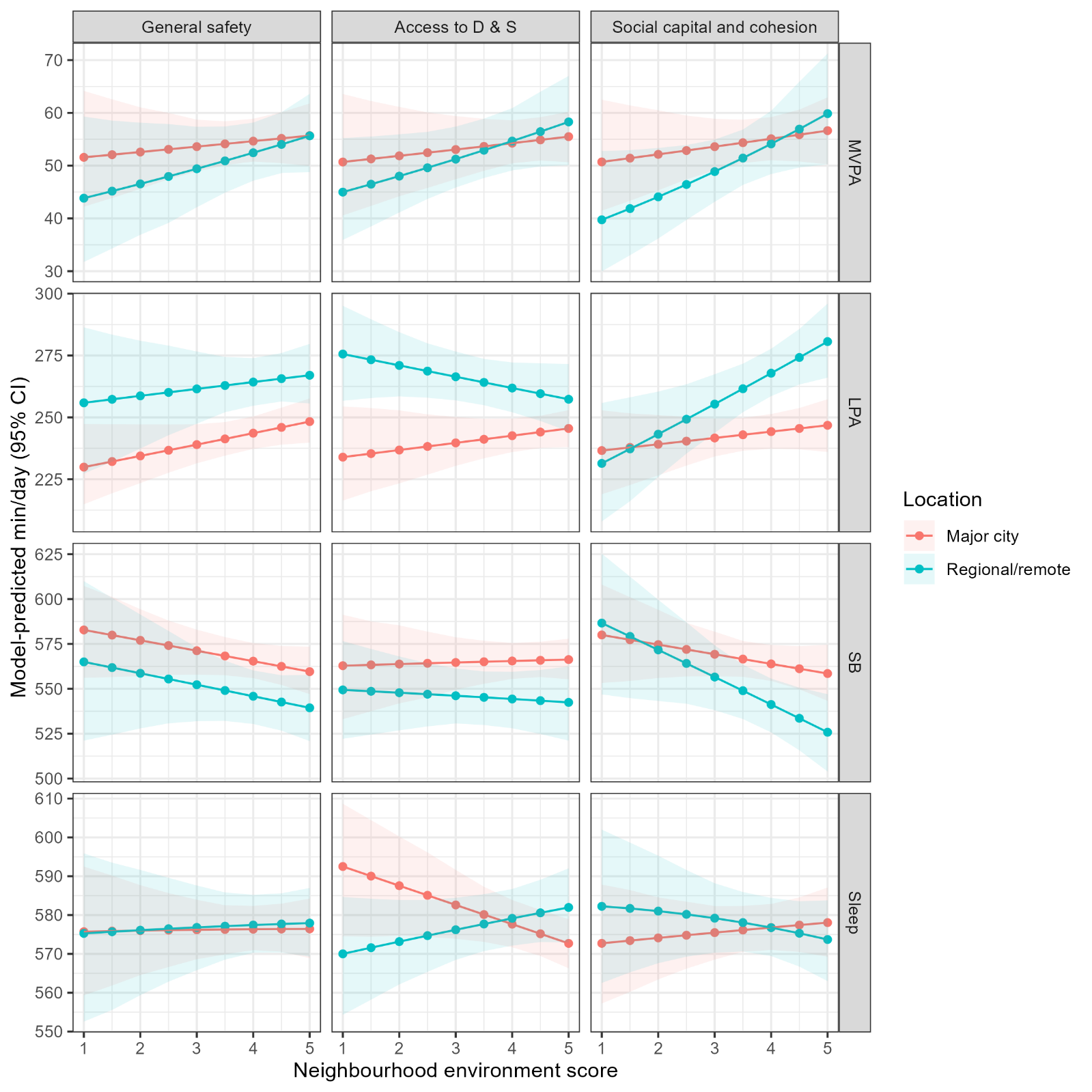


Notes: Access to D & S, access to destinations and services; CI, confidence interval; LPA, light physical activity; MVPA, moderate-to-vigorous physical activity; SB, sedentary behaviour; Analyses were adjusted for age, body mass index, pubertal status, sex, and socioeconomic position
